# Association Between a Capitated, Low-cost, County-Based Public Health Insurance Option and Affordable Care Act Premium Growth in California

**DOI:** 10.1001/jamahealthforum.2023.0488

**Published:** 2023-04-21

**Authors:** Arjun Teotia, Daniel R. Arnold, Richard M. Scheffler

**Affiliations:** 1 School of Public Health, University of California, Berkeley, California; 2 Goldman School of Public Policy, University of California, Berkeley, California

## Abstract

**Importance:**

Empirical evidence is needed on how a capitated, risk-based county plan performs as a viable public option in the Affordable Care Act (ACA) Marketplace in California.

**Objective:**

To estimate whether LA Care—a capitated, county-based public option and California’s largest public insurer—was associated with health insurance premium growth in the Los Angeles (LA) regions of Covered California (CC), the ACA exchange in California.

**Design, Setting, and Participants:**

This economic evaluation used ACA silver plan premium data within the 19 CC regions. Difference-in-differences and event study models used data on plan-level premiums from Health Insurance Exchange Compare for years 2014 to 2022 to estimate the association between LA Care and ACA premium growth in LA.

**Exposures:**

The intervention was LA Care becoming the lowest-cost health plan on the ACA exchange in 2018. The treatment group included the East and West LA regions, and the control group included the remaining 17 CC regions.

**Main Outcomes and Measures:**

The main outcome variable was annual premium growth of plans on CC from 2014 to 2022.

**Results:**

Using 504 plan-level observations for 2014 to 2022, ACA premium growth in LA declined by 4.8% after LA Care became the lowest-cost health plan on the exchange in 2018 (coefficient estimate, −0.048; SE, 0.022; 95% CI, −0.093 to −0.002). Savings due to lower premium growth from 2019 to 2022 were calculated to be $345 million, with approximately 70% of the savings ($242 million) going to the federal government.

**Conclusions and Relevance:**

In this economic evaluation, LA Care was associated with lower premium growth of other health insurance plans in the LA regions of CC, with the majority of savings going to the federal government. California could have captured these savings if it had applied for and received a State Innovation Waiver under section 1332 of the ACA. LA Care may be a viable public option with the potential to be expanded across California through the state’s 16 other county-based health plans.

## Key Points

**Question:**

Is a low-cost, county-based public insurance option associated with slower premium growth on the California Affordable Care Act exchange?

**Findings:**

In this economic evaluation using difference-in-differences and event study models with 504 plan-level observations, LA Care—a low-cost, county-based health plan in Los Angeles—was associated with a 4.8% reduction in Affordable Care Act premium growth in Los Angeles.

**Meaning:**

These findings suggest that adding a county-based public insurance option can be a useful approach to reducing the growth of premiums without the government mandating participation or price regulation to implement a public option.

## Teaser

This economic evaluation estimates the association between a capitated, public insurance option and premium growth in California’s Affordable Care Act exchange.

## Introduction

On Covered California (CC), the state-run Affordable Care Act (ACA) exchange in California, gross annual premiums for members have increased by 41% since CC started in 2014 ($9612 in 2022 vs $6804 in 2014).^1^ Through CC, individuals and their families can purchase private health insurance plans. Exchange coverage is generally intended for those who do not have access to health insurance through their employer, Medicare, or Medicaid. Nearly 90% of CC enrollees receive subsidized coverage in the form of reduced (often 0) premiums and reduced cost sharing.^1^ California is one of the few states that uses an active purchaser model for its exchange, which allows it to standardize benefits and cost sharing, selectively contract with insurers, and negotiate premiums.^2^ Premiums and insurers vary across the 19 regions of CC. Los Angeles (LA) has some of the lowest premiums and premium growth rates on CC. It is also the only region with a public plan—LA Care—which competes with 6 private insurers on the exchange.

**Figure 1.**
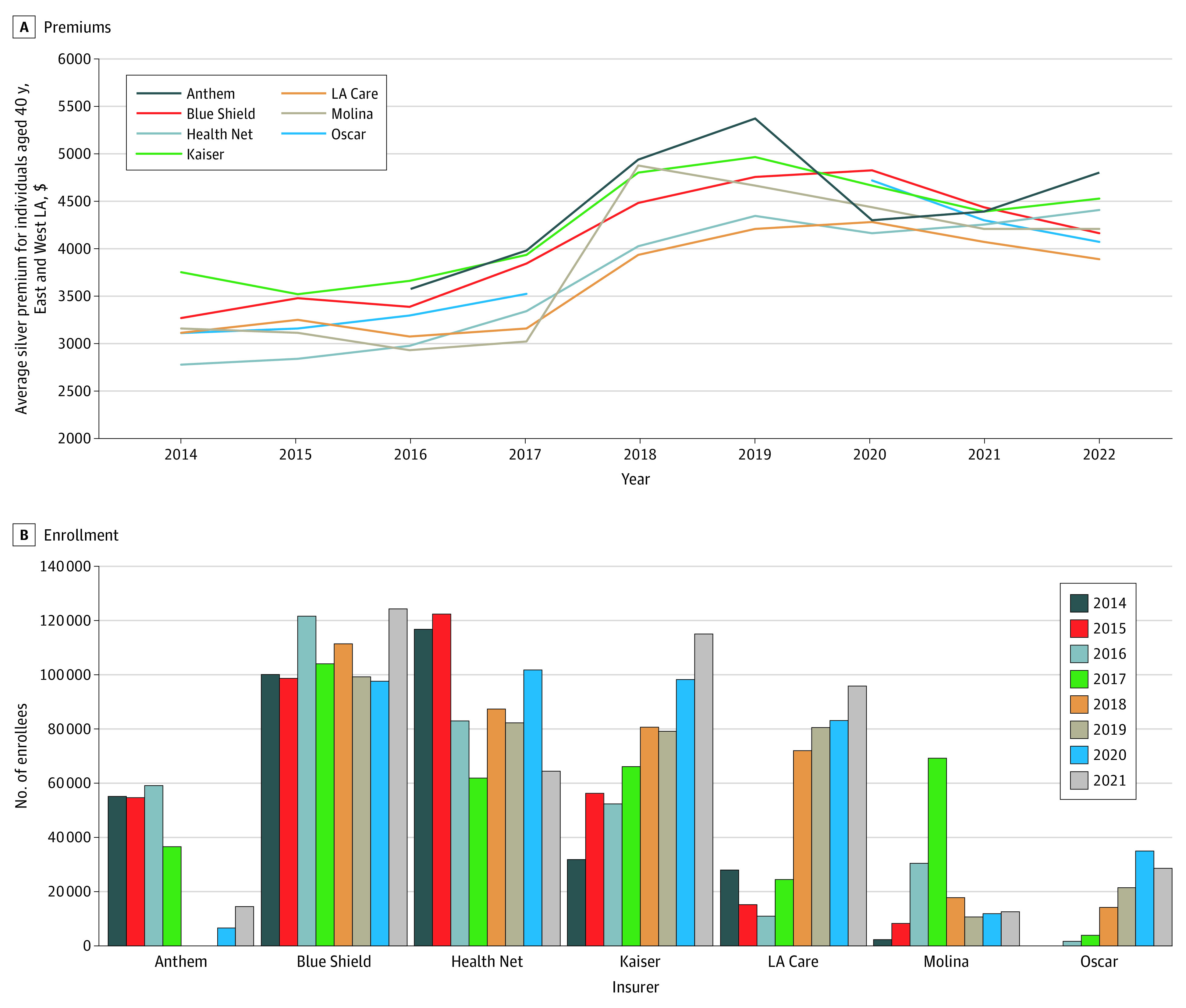
Premiums and Enrollment in Los Angeles (LA), 2014-2022 Data are from Health Insurance Exchange Compare and Covered California.

LA Care is a nonprofit public agency that provides health care access to its 2.7 million members in East and West LA (regions 15 and 16 of CC). Established in 1997, LA Care entered the ACA exchange in 2014, competing against 5 established commercial plans. Being a nonprofit and public plan, it operates on lower margins than its competitors and offers some of the lowest premiums in the region. It is a capitated plan that contracts with physician groups for professional risk and uses contracts with hospitals. Through professional risk contracts, physician groups receive a capitated amount and then assume any risk of their patients needing more services than expected. In 2018, LA Care offered the lowest premium in both LA regions for the first time since the exchange opened in 2014 (Figure 1A). A lower premium can increase consumer welfare given that the low premium is not due to narrower networks, fewer benefits, or a favorable risk profile. LA Care has a 3-star quality rating and offers 4 tiers of plans: platinum, gold, bronze, and silver.^3^ LA Care has a wide network of hospitals, with 57 in-network hospitals. It has also been successful in terms of gaining enrollment. From 2014 to 2017, LA Care had approximately 25 000 enrollees per year. In 2018, when it became the lowest-cost plan, enrollment increased to 74 000 (Figure 1B). Most of this new enrollment came from Molina Healthcare (62%) and Anthem Blue Cross (22%). Switching from Molina (2-star quality rating) to LA Care was associated with consumers benefiting from a lower-cost, higher-quality plan through LA Care. LA Care has not only succeeded in retention but also experienced increased enrollment to 115 000 in 2022. Thus, our primary question is whether LA Care’s low premiums are associated with lower growth of ACA premiums in LA compared with the rest of California.

The effect of insurer competition on ACA premiums and premium growth is well documented in the literature. Most previous literature has focused on how an additional exchange insurer would influence ACA premiums. Dafny et al^4^ provided one of the most compelling pieces of evidence using UnitedHealthcare’s decision to not participate in any of the state- or federally run ACA exchanges. The authors estimated that the second-lowest silver premiums would have been 5.4% lower on average in the first year of the exchanges if UnitedHealthcare had chosen to participate. They further showed that premiums would have been 11.1% lower in 2011 if all active insurers in each state’s insurance market had participated in all rating areas of the state’s exchange. Lissenden^5^ built on the work of Dafny et al,^4^ finding that an additional insurer decreased premium plus cost-sharing parameters by 5%. Abraham et al^6^ found that each additional insurer on the exchanges was associated with a 4% decrease in premiums. Polyakova et al^7^ found that an increase in the number of insurers from the 10th to 90th percentile was associated with a 9% decrease in annual second-lowest silver premiums.

Our work extends this research and is the first known study to empirically evaluate how a county-based public option performs in the ACA Marketplace in California. Specifically, we statistically evaluated whether LA Care was associated with reduced premium growth in LA compared with premium growth in other regions of CC.

## Methods

For this economic evaluation, our primary data on ACA premiums in California are from the Robert Wood Johnson Foundation’s Health Insurance Exchange (HIX) Compare and CC for 2014-2022. Health Insurance Exchange Compare contains comprehensive plan-level information on the metal level (platinum, gold, silver, and bronze), rating area, and premiums for nearly every ACA-compliant individual and fully insured small group market in the US. Because HIX Compare did not report premiums for 2014, we augmented our data for 2014 with data from CC. Premiums vary across regions, insurers, and over time. We focused on the annual premium for silver plans, as 58% of all plans sold on CC are silver plans. Our data set has 504 observations, with the main variable of interest being annual premium growth. Because the study uses secondary data on premiums and is nonhuman research, it was exempted from review by the University of California, Berkeley, institutional review board. The study follows the Consolidated Health Economic Evaluation Reporting Standards (CHEERS) reporting guideline.

### Premium Growth Rates and LA Care

We estimated the association of LA Care with premium growth using a difference-in-differences model. The intervention in our setting is LA Care becoming the lowest-cost plan in 2018. Our treatment group was private insurers in LA since 2019 (excluding LA Care) 1 year after LA Care became the lowest-cost plan. The control group consisted of plans in the 17 non-LA regions of CC. We estimate the following equation:

γ*_prt_* = *β*_0_ + *β*_1_
*Post_t_ × Treatment_r_* + *P_p_* + *R_r_* + *T_t_* + *Ε_prt_*.

Our main outcome variable, γ*_prt_*, is premium growth. Following Dafny et al,^8^ we focused on premium growth rates instead of premium levels to reduce concerns related to time-invariant differences in the risk profiles of plans or other characteristics that might be correlated with premium levels. Our treatment effect is the coefficient on *Post_t_ × Treatment_r_*, β_1_. The control group contained 17 CC regions. We included plan, region, and year fixed effects in the model, denoted by *P_p_*, *R_r_*, and *T_t_*, respectively. The inclusion of year fixed effects captures mean changes in premium growth across years, region fixed effects captures differences in mean growth rates across markets, and plan fixed effects captures differences in mean growth rates across plans. The SEs were clustered at the region level, which accounted for serial correlation within regions.^9^

Next, we estimated the dynamic association between LA Care and premium growth through an event study analysis.^10^ The event was defined as LA Care offering the lowest premium. As becoming the lowest-cost plan is not an exogenous event in itself, our event study shows a time series comparison of premiums between LA and non-LA counties. Using time as the independent variable, the event study estimated the event's association with premium growth at each 1-year window.

### Statistical Analysis

To validate our difference-in-differences model, we tested for parallel trends. Parallel trends imply that there are no significant differences in premium growth trends between treatment and control groups prior to 2019. In other words, had LA Care not had the lowest premium in 2018, both LA and non-LA regions would have followed the same premium growth path. First, we used a formal *F* test to test for parallel trends. The *F* test compares the fit of premium growth trends for treatment and control regions. The null hypothesis for the test is that trends are parallel. Second, we plotted trends in premium growth in treatment and control regions prior to LA Care being the lowest-cost plan on the market. To test the significance of the regression coefficient in our main analysis, we used a *t* test. We calculate the *t* statistic by dividing the estimated coefficient by its SE. The null hypothesis of 0 coefficient is rejected if the *t* statistic is greater than 1.96. The threshold for significance was set at a 2-sided *P* < .05.

## Results

Table 1 shows the mean annual premium and premium growth for LA and non-LA regions (17 CC regions). Mean (SD) premium growth in CC was 1.38% (9.89%) for our sample. Premiums in LA were lower than non-LA premiums for all years of our sample. The mean (SD) premium in LA was $4269 ($672) before 2019 and $4758 ($640) since 2019. Mean (SD) premiums in the 17 other non-LA regions were $5620 ($1112) before 2019 and $6696 ($1840) since 2019. Premium growth was similar in both regions before 2019 (LA, 7.19%; non-LA, 8.00%), while premiums in LA fell by approximately double that of non-LA regions since 2019 (−6.00% for LA vs −3.67% for non-LA). This descriptive evidence implies that in addition to having lower premiums, LA also had lower premium growth since 2019. Between 2018 and 2022, changes in the percentage of enrollees in silver plans did not change the results (treated regions, 234 460 enrollees [61%] in 2018 vs 298 150 [60%] in 2022; control regions, 537 650 enrollees [54%] in 2018 vs 684 750 [58%] in 2022).

**Table 1.** Premiums and Premium Growth in Covered California^a^

Year	Premium, mean (SD), $		Premium growth, %	
	LA	Non-LA	LA	Non-LA
2014	3966 (532)	5044 (661)	NA	NA
2015	3975 (415)	5205 (687)	0.21	3.18
2016	3971 (478)	5248 (713)	−0.10	0.83
2017	4260 (542)	5790 (1020)	7.27	10.32
2018	5171 (569)	6812 (1302)	21.39	17.65
2019	5304 (526)	7118 (1574)	2.58	4.50
2020	5083 (525)	7000 (1750)	−4.17	−1.66
2021	4642 (347)	6870 (2050)	−8.68	−0.86
2022	4004 (146)	5795 (1697)	−13.75	−15.65
Average (2014-2018)	4269 (672)	5620 (1112)	7.19	8.00
Average (2019-2022)	4758 (640)	6696 (1840)	−6.00	−3.67

Abbreviations: LA, Los Angeles; NA, not applicable.

^a^ Data are from Health Insurance Exchange Compare and Covered California from 2014 to 2022 (504 observations). The unit of observation is a silver plan offered on Covered California. Premiums are adjusted for annual Consumer Price Index and presented in 2022 dollars. All statistics are unweighted. Premium growth = 100 × (current year premium – previous year premium) / previous year premium.

Next, we investigated whether the low premium growth in LA was associated with the low cost of LA Care. Table 2 shows the results from the regression analysis. Relative to the rest of California, ACA premium growth in LA was 4.8% lower since 2019 after LA Care became the lowest-cost plan (coefficient estimate, −0.048; SE, 0.022; 95% CI, −0.093 to −0.002). The coefficient is statistically significant at a 5% level. Our results show that LA Care was associated with lower premium growth in LA. After conducting tests for linear pretrends, both graphic and formal tests indicated that parallel trends were satisfied, which supports the validity of our regression model. Given that mean (SD) premiums in LA were $4758 ($640) after treatment, we calculated annual savings from LA Care to be $225 per enrollee in LA. As the annual enrollment in LA in 2019 was 342 840 (eTables 1 and 2 in Supplement 1), the low premium growth of LA Care led to an estimated $77 million in annual savings in 2019 (eAppendix in Supplement 1). Similarly, savings from lower premium growth in LA was $85 million in 2020, $88 million in 2021, and $95 million in 2022, totaling $345 million over 2019 to 2022. Approximately 70% of the savings ($242 million) went to the federal government.

**Table 2.** Association of LA Care With Premium Growth^a^

	Premium growth, coefficient estimate (SE) [95% CI]
Average treatment effect	−0.048 (0.022) [−0.093 to −0.002]
No. of observations	504
*R* ^2^	0.78
Fixed effects	
Region	Yes
Plan	Yes
Year	Yes

^a^ Data are from Health Insurance Exchange Compare and Covered California from 2014 to 2022. The unit of observation is a silver plan offered on Covered California. SE is clustered by region. The *F* statistic for parallel trends = 2.55 (parallel trends are satisfied). *P* = .04; *t* = −2.21. All statistics are unweighted.

**Figure 2.**
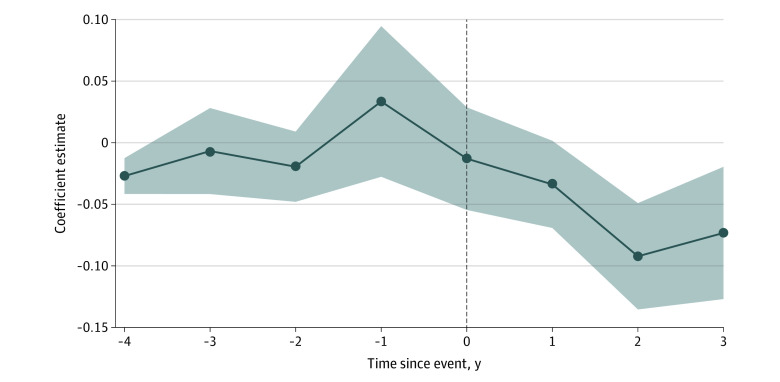
Event Study of the Association Between LA Care and Affordable Care Act Premium Growth Data are from Health Insurance Exchange Compare and Covered California. The shaded region shows 95% CIs. Event year 0 = 2019.

Figure 2 displays the results of the event study. We evaluated 4 windows before and 4 windows after the event. The x-axis shows years from the event (2018 = −1; 2019 = 0; 2020 = 1), the y-axis shows premium growth, and the shaded region shows 95% CIs. Prior to the event in 2019, premium growth was approximately 0, with CIs distributed on either side of 0. One year after the event, the correlation was negative and statistically significant (coefficient estimate, −0.034; 95% CI, −0.067 to −0.001). For all years since 2019, the upper CIs were less than 0, showing a significant decline in premium growth.

To test the robustness of these results, we conducted various placebo tests (Table 3). The first 2 rows show the placebo estimates after changing the timing of treatment to 2017 (placebo 1) and 2018 (placebo 2), respectively. In both cases, the coefficient estimate was statistically insignificant (placebo 1, −0.044 [95% CI, −0.089 to 0.001]; placebo 2, −0.036 [95% CI, −0.086 to 0.014]). The third through fifth rows show the placebo estimates after changing the region of treatment to northern counties (placebo 3), northern counties and North Bay counties (placebo 4), and northern counties, North Bay counties, and Sacramento (placebo 5), respectively. Again, we found no significant coefficient estimate on premium growth in these samples. This lack of a placebo effect supports our earlier results that LA Care, a low-cost public option, was significantly associated with slower commercial premium growth in the LA region of CC by 4.8% over 2019 to 2022.

**Table 3.** Placebo Estimates of the Correlation Between LA Care and Premium Growth^a^

	Premium growth, coefficient estimate (SE) [95% CI]
Placebo 1 (treatment year = 2017)	−0.044 (0.021) [−0.089 to 0.001]
Placebo 2 (treatment year = 2018)	−0.036 (0.024) [−0.086 to 0.014]
Placebo 3 (treatment region = northern counties)	−0.029 (0.018) [−0.009 to −0.068]
Placebo 4 (treatment region = placebo 3 + North Bay)	−0.046 (0.053) [−0.065 to 0.157]
Placebo 5 (treatment region = placebo 4 + Sacramento)	−0.045 (0.043) [−0.046 to 0.135]

^a^ Data are from Health Insurance Exchange Compare and Covered California from 2014 to 2022. The unit of observation is a silver plan offered on Covered California. Region, year, and plan fixed effects were used in the analysis. SEs are clustered by region. All statistics are unweighted.

## Discussion

Colorado, Nevada, and Washington have already enacted legislation to make insurance markets more competitive by adding a public option to the ACA exchange.^11^ A few states, including Minnesota,^12^ Oregon,^11^ and Vermont,^11^ are also interested in implementing a state-run plan. The legislation requires participation and/or price regulation. California already has a public option, LA Care, that has made its exchange more competitive. We estimated LA Care’s association with ACA premium growth and found a 4.8% decline in premium growth in LA, leading to $345 million in savings through premium growth reduction from 2019 through 2022. LA Care has also successfully increased its exchange enrollment over time, with more than 115 000 enrollees at last count in 2022. This gain in enrollment paired with a reduction in premium growth suggests that LA Care may be a successful county public option for health care insurance. LA Care is 1 of 17 county-based plans in California, so the estimated competitive impact of LA Care in LA could presumably be extended to other regions of the state. Another county plan, CalOptima, has applied to be listed on the ACA exchange. CalOptima is the largest insurer in Orange County, with 911 000 Medi-Cal members who have access to a network of more than 10 600 primary care physicians and specialists, as well as 34 community health centers and 41 acute care and rehabilitation hospitals. Other noncounty plans can also reduce premium growth if they offer a lower price. LA Care uses capitation, has lower administrative costs, and does not have to make a profit, but any plan (public option or not) could reduce premium growth if it offered a low price. The lesson for California is that 70% of the $345 million in savings due to premium growth reductions went to the federal government. A significant portion of the savings to the federal government could have been captured by California if it had applied for and received a State Innovation Waiver under section 1332 of the ACA from the US Department of Health and Human Services. Section 1332 waivers offer states the opportunity to provide innovative health coverage and affordability solutions that decrease costs to the federal government without diminishing coverage. The Department of Health and Human Services recently approved a section 1332 waiver for Colorado’s public option law.^13^

### Limitations

Our study has some limitations. First, as is the case with all difference-in-differences studies, our results hinge on the assumption that there was not another event in the LA region of CC that happened in 2018 that also would have led to lower premium growth. For instance, if the economy in LA declined after 2018 relative to the rest of California, we would expect premiums to potentially not grow as fast due to a reduction in demand. We do not have any such event in mind, but it is a concern that another event could have occurred simultaneously with the intervention being studied. Second, to the extent that a change in the lowest-cost premium reduces the second-lowest-cost premium in a region (thereby reducing the subsidy for all eligible enrollees), it is possible that some enrollees downgraded metal tiers and changed the risk pool for silver plans. We calculated the change in the percentage of enrollees in silver plans in treated vs control regions and found minimal change over time. Treated regions decreased from 61% in 2018 to 60% in 2022, whereas the percentage of enrollees in silver plans in the control regions increased from 54% to 58%. Although these changes are small in magnitude, they are still a limitation of our study. The way subsidies are set up on the exchanges keeps enrollment in silver plans fairly stable. Third, our analysis is limited to California, which, unlike most states, requires standardized benefits and cost sharing and limits the number of plans an insurer can offer on a tier within a rating region. By making plan selection easier, it is possible that there is more shopping in California than in most other states, which would increase the switching rate in California relative to other states. Insurers are more likely to lower premiums when they know customers may switch to the lowest-cost plan.^14^

## Conclusions

This economic evaluation provides the first evidence of an association of a county public option with ACA premium growth in California. We estimated that LA Care was associated with 4.8% lower premium growth in the LA region of CC, producing an estimated $345 million in savings from 2019 to 2022. This evidence is important at a time when several states are considering creating a public option. The study’s findings are important because they show that a county-based public option can be a useful approach in reducing the growth of premiums without the government mandating participation or price regulation to implement a public option.

## Notes

Supplement 1. **eTable 1.** Total Enrollment for Each Region of CC (2014-2022) **eTable 2.** Enrollment Per Insurer in CC (2022) **eAppendix.** Savings

Supplement 2.
Data Sharing Statement

## References

[1] California’s health benefit exchange. Covered California. Accessed October 1, 2022. https://hbex.coveredca.com/data-research/

[2] Weinberg M, Kallerman P. A Study of Affordable Care Act Competitiveness in California. Bay Area Council Economic Institute; 2017. Accessed October 1, 2022. http://www.bayareaeconomy.org/files/pdf/CA_ACA_Report221017.pdf

[3] Quality ratings. Covered California. Accessed November 20, 2022. https://coveredca.com/support/getting-started/quality-ratings/

[4] Dafny L, Gruber J, Ody C. More insurers lower premiums: evidence from initial pricing in the health insurance marketplaces. Am J Health Econ. 2015;1(1):53-81. doi:10.1162/ajhe_a_00003

[5] Lissenden B. Three’s a crowd? the effect of insurer participation on premiums and cost-sharing parameters in the initial years of the ACA Marketplaces. Am J Health Econ. 2017;3(4):477-506. doi:10.1162/ajhe_a_00085

[6] Abraham JM, Drake C, McCullough JS, Simon K. What drives insurer participation and premiums in the Federally-Facilitated Marketplace? Int J Health Econ Manag. 2017;17(4):395-412. doi:10.1007/s10754-017-9215-y 28447230

[7] Polyakova M, Bundorf MK, Kessler DP, Baker LC. ACA Marketplace premiums and competition among hospitals and physician practices. Am J Manag Care. 2018;24(2):85-90. 29461855

[8] Dafny L, Duggan M, Ramanarayanan S. Paying a premium on your premium? consolidation in the US health insurance industry. Am Econ Rev. 2012;102(2):1161-1185. doi:10.1257/aer.102.2.1161 29521483

[9] Bertrand M, Duflo E, Mullainathan S. How much should we trust differences-in-differences estimates? Q J Econ. 2004;119(1):249-275. doi:10.1162/003355304772839588

[10] De Chaisemartin C, d’Haultfoeuille X. Two-way fixed effects estimators with heterogeneous treatment effects. Am Econ Rev. 2020;110(9):2964-2996. doi:10.1257/aer.20181169

[11] Monahan CH, Govannelli J, Lucia K. Update on state public option-style laws: getting to more affordable coverage. *To the Point* blog. March 29, 2022. Accessed March 16, 2023. https://www.commonwealthfund.org/blog/2022/update-state-public-option-style-laws-getting-more-affordable-coverage

[12] Deng G. House lawmakers advance a bill creating a MinnesotaCare public option for all state residents. *Minnesota Reformer*. February 8, 2023. Accessed March 16, 2023. https://minnesotareformer.com/briefs/house-lawmakers-advance-a-bill-creating-a-minnesotacare-public-option-for-all-state-residents

[13] Monahan CH, Giovannelli J, Lucia K. HHS approves nation’s first section 1332 waiver for a public option plan in Colorado. *To the Point* blog. July 13, 2022. Accessed October 30, 2022. https://www.commonwealthfund.org/blog/2022/hhs-approves-nations-first-section-1332-waiver-public-option-plan-colorado

[14] Gabel JR, Arnold DR, Fulton BD, . Consumers buy lower-cost plans on Covered California, suggesting exposure to premium increases is less than commonly reported. Health Aff (Millwood). 2017;36(1):8-15. doi:10.1377/hlthaff.2016.0902 28069841

