# Supplementary material for: Association Between a Capitated, Low-cost, County-Based Public Health Insurance Option and Affordable Care Act Premium Growth in California
Source: JAMA Health Forum. 2023 Apr 21;4(4):e230488. doi: 10.1001/jamahealthforum.2023.0488 (PMC10122165; doi:10.1001/jamahealthforum.2023.0488)
Supplement: Supplement 1. — eTable 1. Total Enrollment for Each Region of CC (2014-2022) eTable 2. Enrollment Per Insurer in CC (2022) eAppendix. Savings [file jamahealthforum-e230488-s001.pdf]

Supplemental Online Content

Teotia A, Arnold DR, Scheffler RM. Association between a capitated, low-cost, county-based public health insurance option and Affordable Care Act premium growth in California. *JAMA Health Forum*. 2023;4(4):e230488. doi:10.1001/jamahealthforum.2023.0488

eTable 1. Total Enrollment for Each Region of CC (2014-2022)
eTable 2. Enrollment Per Insurer in CC (2022)
eAppendix. Savings

This supplemental material has been provided by the authors to give readers additional information about their work.

© 2023 Teotia A et al. *JAMA Health Forum.*

eTable 1: Total Enrollment for Each Region of CC (2014-2022)

Region	Total Enrollment (2014-2022)	Observations
1 – Northern Counties	166,470	18
2 – North Bay Counties	428,190	36
3 – Greater Sacramento	625,510	27
4 – San Francisco County	301,600	36
5 – Contra Costa County	392,630	27
6 – Alameda County	554,450	18
7 – Santa Clara County	390,080	27
8 – San Mateo County	213,920	36
9 – Central Coast - North	135,100	18
10 – Central Valley - North	297,610	27
11 – Greater Fresno Area	243,770	18
12 – Central Coast - South	473,190	18
13 – Eastern Region	22,060	18
14 – Kern County	139,660	27
15 – Los Angeles - East	1,494,490	45
16 – Los Angeles - West	1,524,330	36
17 – Inland Empire	937,510	27
18 – Orange County	917,030	27
19 – San Diego County	880,710	36

Notes: Data used from HIX Compare and Covered California (CC) from 2014-2022 (504 observations). Unit of observation is a silver plan offered on CC.

eTable 2: Enrollment Per Insurer in CC (2022)

Insurer	Median Enrollment (2014-2022)
BlueShield of California	495,400
Chinese Community Health Plan	3,780
Health Net	143,150
Kaiser Permanente	608,790
LA Care Health Plan	115,070
Molina	620
Sharp Health Plan	31,750
Valley Health Plan	19,880
Western Health Advantage	9,880

Notes: Data used from HIX Compare and Covered California (CC) from 2014-2022 (504 observations). Unit of observation is a silver plan offered on CC. All statistics are unweighted.

© 2023 Teotia A et al. *JAMA Health Forum.*

eAppendix. Savings

The net (i.e., after subsidies) monthly premiums per member were $131 and $178 in regions 15 and 16, respectively, in June 2022 while the gross monthly premiums per member were $473 and $499. This implies the federal government covered about 70% of the premium across the two regions in LA in 2022. Specifically, 72% in Region 15 ((473-131)/473) and 64% in Region 16 ((499-178)/499). The federal government's proportion of premiums was also about 70% over the 2019-2021 period.

We used our regression coefficient (-0.048) and the average premium from 2019-2022 ($4758) to calculate the approximate saving per year per enrollee ($225 to the closest multiple of 5). Additionally, we created confidence intervals (CI) for our savings estimate using 90, 95, and 99% CI's of our regression coefficient. Using 90% CI (-0.010) approximates to $50 in savings and the lower CI (-0.085) approximates to $400 in savings. We multiply each of the savings estimate with yearly enrollment for the years 2019 (342,840), 2020 (381,560), 2021 (392,310), and 2022 (426,620). This leads to total approximate savings with a lower bound of $77 million, average savings of $345 million, and an upper bound of $615 million [77 (345) 615]. The range of savings for 95% and 99% CIs are [1 (345) 680] and [-100 (345) 800] respectively. We report $345 million in our main analysis as an average approximation of savings. 70% of these savings went to the federal government ($245 million).

© 2023 Teotia A et al. *JAMA Health Forum.*
