# Supplementary material for: Association Between a Capitated, Low-cost, County-Based Public Health Insurance Option and Affordable Care Act Premium Growth in California
Source: JAMA Health Forum. 2023 Apr 21;4(4):e230488. doi: 10.1001/jamahealthforum.2023.0488 (PMC10122165; doi:10.1001/jamahealthforum.2023.0488)
Supplement: Supplement 2. — Data Sharing Statement [file jamahealthforum-e230488-s002.pdf]

Data Sharing Statement

Teotia. Association Between a Capitated, Low-Cost, County-Based Public Health Insurance Option and Affordable Care Act Premium Growth in California. *JAMA Health Forum*. Published April 21, 2023. doi:10.1001/jamahealthforum.2023.0488

Data
Data available: No

Additional Information
Explanation for why data not available: The data sources used in this study are publicly available. The programming code used to generate these results is available at https://drive.google.com/file/d/19i69kZzXjXUVSmgsZYnVLsq9ERfywZu7/view?usp=sharing
